# The role of m6A RNA methylation in cancer metabolism

**DOI:** 10.1186/s12943-022-01500-4

**Published:** 2022-01-12

**Authors:** Yuanyuan An, Hua Duan

**Affiliations:** Gynecological Mini-Invasive Center, Beijing Obstetrics and Gynecology Hospital, Capital Medical University, Beijing Maternal and Child Health Care Hospital, 17 Qihelou Street, Dongcheng District, Beijing, 100006 China

**Keywords:** The m6A, Cancer, Metabolism reprogramming

## Abstract

Metabolic reprogramming is one of the main characteristics of malignant tumors, which is due to the flexible changes of cell metabolism that can meet the needs of cell growth and maintain the homeostasis of tissue environments. Cancer cells can obtain metabolic adaptation through a variety of endogenous and exogenous signaling pathways, which can not only promote the growth of malignant cancer cells, but also start the transformation process of cells to adapt to tumor microenvironment. Studies show that m6A RNA methylation is widely involved in the metabolic recombination of tumor cells. In eukaryotes, m6A methylation is the most abundant modification in mRNA, which is involved in almost all the RNA cycle stages, including regulation the transcription, maturation, translation, degradation and stability of mRNA. M6A RNA methylation can be involved in the regulation of physiological and pathological processes, including cancer. In this review, we discuss the role of m6A RNA methylation modification plays in tumor metabolism-related molecules and pathways, aiming to show the importance of targeting m6A in regulating tumor metabolism.

**Supplementary Information:**

The online version contains supplementary material available at 10.1186/s12943-022-01500-4.

## Background

One of the hallmarks of cancer is uncontrolled cell proliferation. The important reason is that the cell changes in controlling metabolism and proliferation signaling pathways, in which metabolic changes provide energy and anabolic requirements to enhance cell proliferation. In order to achieve, adapt and maintain the proliferative ability, cancer cells must activate or enhance metabolism pathways [1]. As early as 1924, when it was believed that tumor cells can only obtain energy from protein decomposition and lipolysis, which provides energy for the process of non-stop replication. However, Warburg found that they could rapidly consume glucose under aerobic conditions for activating glycolysis and converting it into lactic acid, which is the best-known Warburg effect [2]. Since then, the metabolic recombination of tumor cells has been widely valued by researchers. In addition, tumor cells induce chronic hypoxia through various mechanisms, causing endothelial dysfunction and promoting tumor progression, which is correlated with the abnormal metabolism of tumor cells. Under the condition of hypoxia, mitochondrial metabolic stress can accelerate the terminal differentiation of T cells, up-regulate the expression of ROS in T cells, promote T cell dysfunction and failure, and promote tumor progression [3]. Metabolic reprogramming is conducive to the development of tumor. The usage of nutrients to produce metabolic precursors for cell anabolism can meet the energy needs of cell maintenance and biosynthesis. Therefore, chemotherapy and immunotherapy targeting metabolism are becoming effective methods for cancer treatment.

Epigenetics is the study of reversible and heritable phenotypes, including DNA and RNA methylation, histone modification, non-coding RNA modification and chromatin rearrangement. At present, as an important branch of epigenetics, more than 100 kinds of RNA chemical modifications have been found [4]. In 1974, a methyl group at the N6 position of adenine was first found in mRNA. This modified base is called m6A, the most abundant internal modification on eukaryotic mRNA. The average 1000 nucleotides contain 1–2 m6A residues [5]. M6A mainly appears in the RRACH sequences, and is significantly enriched near the stop codon, 3’-UTR and long intron [6]. The basic processes of m6A modification is that it is installed by m6A methyltransferase, removed by m6A demethylase and recognized by m6A reading molecules thus regulating RNA metabolism. More and more evidences show that m6A can influence the expression of target genes, thus regulating a variety of physiological processes, including self-renewal, invasion and proliferation. In the molecular mechanism, m6A is involved in almost all RNA metabolism processes, including translation, degradation, splicing, exporting and folding [7]. In recent years, many studies show that m6A is widely involved in tumor regulation, which further regulates the occurrence and development of tumor by regulating tumor metabolism.

## Cancer metabolism

For many years, the consensus of medical community for tumors is that they are gene-related diseases, almost all cancers are caused by gene changes. Accumulating evidences of cancer phenotypes indicate that all cancers share six biological abilities in the process of multi-step development: continuous proliferation signaling, escape from proliferation inhibitor, resistance to cell death and apoptosis, immortality of replication, induction of angiogenesis, and promotion of invasion and metastasis [8]. However, follow-up studies have shown that cancer has two other typical characteristics: reprogrammed energy metabolism and avoidance of immune-mediated destruction [9]. Therefore, many people think that tumor is essentially a metabolic disease. Even in the same patient or experimental model, primary and metastatic tumor cells have different metabolic characteristics. Although tumors are caused by mutations in oncogenes and tumor suppressor genes, these mutations can directly regulate the expression and activity of metabolic enzymes. In tumor, metabolic recombination is a necessary condition to maintain vitality and function of cancer cells, and also a key factor to regulate tumor immune escape. Recently, studies found that metabolic reprogramming of tumor cells is one of the important markers of cancer cells growth and resistance to chemotherapy [10, 11]. The imbalance of glucose metabolism, fatty acid synthesis and glutamine decomposition are closely related to the malignant biological behaviors of cancer cells, including proliferation, drug resistance, invasion and metastasis. Tumor metabolism is closely related to the expression of a variety of signaling pathways, transcription factors and metabolism-related enzymes. In addition, a variety of tumor-related signaling pathways participate in the regulation of tumor metabolism. In order to survive and adapt to these severe environmental pressures, cancer cells change their metabolic pathways to capture external metabolites and maximize the efficiency of metabolic enzymes [12]. Under the condition of sufficient oxygen, normal cells are mainly supplied with energy of OXPHOS, which changes glucose into pyruvate in the cytoplasm, then enters mitochondria to produce CO_2_ and energy through tricarboxylic acid cycle. When oxygen is insufficient, the energy is mainly supplied by glycolysis, and pyruvate changes into lactic acid in the cytoplasm. It is found that compared with normal cells, tumor cells absorb more glucose, but use less glucose through OXPHOS. Even under the condition of sufficient oxygen, they mainly rely on glycolysis pathway for energy supply, which is called Warburg effect. However, later researchers found that tumor cells have biphasic metabolism, which may be the reason of chemotherapy resistance of tumor cells, known as anti-Warburg effect or metabolic coupling [13]. What is biphasic metabolism? Under normal conditions, tumor cells show glycolytic phenotype. However, when glucose supply is reduced and lactic acid accumulation leads to lactic acidosis, tumor cells will inhibit the activity of glycolytic enzymes and eventually reduce glycolysis, showing a non-glycolysis phenotype, transforming to OXPHOS to maintain the proliferation of tumor cells [14, 15]. This suggests that metabolic recombination in tumor cells is a very complex process, which is based on promoting the growth and progression of tumor cells and maintaining dynamic balance. Therefore, the abnormal expression of signaling pathways, signaling molecules and metabolic enzymes can promote the metabolic reorganization of tumor cells.

## The m6A

The most common mRNA modification in mammals is m6A modification, which is composed of 0.1–0.4% adenylate residues, especially at the beginning of 3’-UTR near the translation stop codon, the common sequence 5’-RRACH-3’ is usually embedded [16, 17]. M6A modification can regulate all stages of RNA cycle, such as the processing, degradation, nuclear export and translation of RNA, thus regulating RNA expression and function, which also including a variety of non-coding RNAs [18–21]. The modification of m6A is dynamic and reversible, and plays a biological role mainly mediated by “writers”, “erasers” and “readers”. In tumor, m6A RNA methylation is a double-edged sword. Current studies suggest that the regulation of target genes by m6A and its effect on tumor progression depends on three factors: the target is oncogene or tumor suppressor gene; the abnormal level of m6A in cancer mainly depends on the expression and activity of “writers” and “erasers”; target mRNA is regulated after modification, which is mainly determined by “readers”.

At present, the researches on m6A “writers” is the most extensive. The “writers” of m6A are mainly composed of METTL3, METTL14 and their cofactor WTAP (Table 1). There is an S-adenosylmethionine binding motif in METTL3 and METTL14. These two genes are located in the nuclear spot together and formed a stable heterodimer complex in the ratio of 1:1 [22]. As a pseudo-methyltransferase, METTL14 plays an important role in stabilizing METTL3 and recognizing target RNAs. As the main regulatory and component molecule of m6A methylation complex, WTAP can help METTL3 and METTL14 locate in the nuclear plaques. In addition, m6A “writers” also include METTL16, KIAA1429 and RBM15. Among them, KIAA1429, as the largest scaffold component of m6A methyltransferase complex, is used to regulate 3’-UTR of genes and m6A methylation near the stop codon [23, 24].

**Table 1:** The function of m6A methylation enzymes in RNA metabolism

Type	Factor	Full name	Function
M6A Writers	METTL3	Methyltransferase-like 3	Catalyzes m6A modification
	METTL14	Methyltransferase-like 14	A core subunit of m6A methyltransferase, form heterodimer with METTL3 to catalyze m6A modification
	WTAP	Wilms tumor 1- associated protein	Regulatory subunit of m6A methyltransferase and recruits METTL3 and METTL14 into the nuclear speckles
	VIRMA (KIAA1429)	Vir-like m6A methyltransferase associated	Recruits METTL3 and METTL14 and guides m6A methylation at specific sites to facilitate m6A installation, thus inducing mRNA splicing and RNA processing
	METTL16	Methyltransferase-like 16	Catalyzes m6A modification
	RBM15	RNA binding motif protein 15	Directs METTL3-METTL14 heterodimer to specific RNA sites
	RBM15B	RNA binding motif protein 15B	Directs METTL3-METTL14 heterodimer to specific RNA sites
M6A Erasers	FTO	Fat mass and obesity-associated	Acts as m6A demethylase to promote mRNA splicing and translation
	ALKBH5	AlkB homologue 5	Removes m6A modification to promote mRNA nuclear processing and mRNA export
	ALKBH1	AlkB homologue 1	Removes m6A modification to act as tRNA demethylase by removing N(1)-methyladenine, regulating translation initiation and elongation
M6A Readers	YTHDF1	YTH N6-methyladenosine RNA binding protein 1	Promotes mRNA translation initiation
	YTHDF2	YTH N6-methyladenosine RNA binding protein 2	Promotes mRNA degradation
	YTHDF3	YTH N6-methyladenosine RNA binding protein 3	Interacts with YTHDF1 to promote mRNA translation or interacts with YTHDF2 to promote mRNA degradation
	YTHDC1	YTH domain containing 1	Promotes mRNA splicing and transcriptional silencing
	YTHDC2	YTH domain containing 2	Improves the translation efficiency of target mRNA
	eIF3	Eukaryotic translation initiation factor 3 subunit A	Promotes mRNA translation
	IGF2BP1/2/3	Insulin-like growth factor 2 mRNA binding protein 1/2/3	Promotes the stability and translation of mRNA
	HNRNPA2B1	Heterogeneous nuclear ribonucleoprotein A2/B1	Promotes primary miRNA processing and mRNA splicing

Demethylation is mainly acted by FTO and ALKBH5. As a reversible step of m6A methylation, demethylase FTO can regulate fat production and energy homeostasis [25]. ALKBH5 has the highest expression in testis, but lower expression in heart and brain, which can affect nuclear RNA output, metabolism and gene expression [26].

Besides “writers” and “erasers”, another indispensable group in m6A is called “readers”. They can recognize modifications and combine with them. Different readers can perform different biological functions. The most famous m6A readers are YTHDF family and IGF2BP family [27]. The YTH domains in human cells, including YTHDF1-3 and YTHDC1-2, preferentially bind to the m6A modified RNA in RRACH common sequence. YTHDF2 is the most widely studied m6A reader. Under normal or stress conditions, it promotes the degradation of m6A dependent RNA [28]. YTHDF1 binds to the m6A site around the stop codon and enhances the efficiency of RNA translation by interacting with eIF3. Unlike YTHDF family, which mainly participate in regulating the splicing and translation of pre-mRNA, “readers” from IGF2BP family is responsible for recruiting RNA stabilizers to promote mRNA stability, thereby affecting tumor progression [29].

## The m6A and metabolic signaling pathways

The development of tumor progression needs metabolism reprogramming. In fact, tumor metabolism reprogramming is mainly composed of bioenergy metabolism and biosynthesis metabolism. Bioenergy metabolism is mainly mediated by mitochondrion, while the biosynthesis metabolism is mainly refers to regulation the synthesis of glucose, lipid and amino acid. In addition to the tumor cells, the other cells in tumor microenvironment including endothelial cells, fibroblasts and immune cells also need to undergo metabolic reorganization to meet the needs of promoting tumor progression. Tumor related signaling pathway is a series of enzymatic reaction pathways with various effects on tumor cells. Thousands of genes can regulate tumor progression, but most of them are attributed to the activation or inhibition of tumor-related signaling pathways eventually. For tumor metabolism reprogramming, it is found that different metabolism may be concentrated to the same signaling pathway, but play distinct roles. Therefore, comprehensive analyze the action modes of different metabolism in the same signaling pathway can comprehensively understand the impact of metabolic reprogramming on tumor progression. In the previous review, Wang et al. reported that the complex role of m6A methylation in tumor progression in detail, which focused on the potential role of m6A methylation in distinct tumors [30]. However, in our review, we focused on study the role of m6A methylation in cancer metabolism, including glucose, amino acid and fatty acid metabolism, which also have been extended to the metabolism-related pathways and transcription factors.

### The m6A and mTOR

**Fig. 1:**
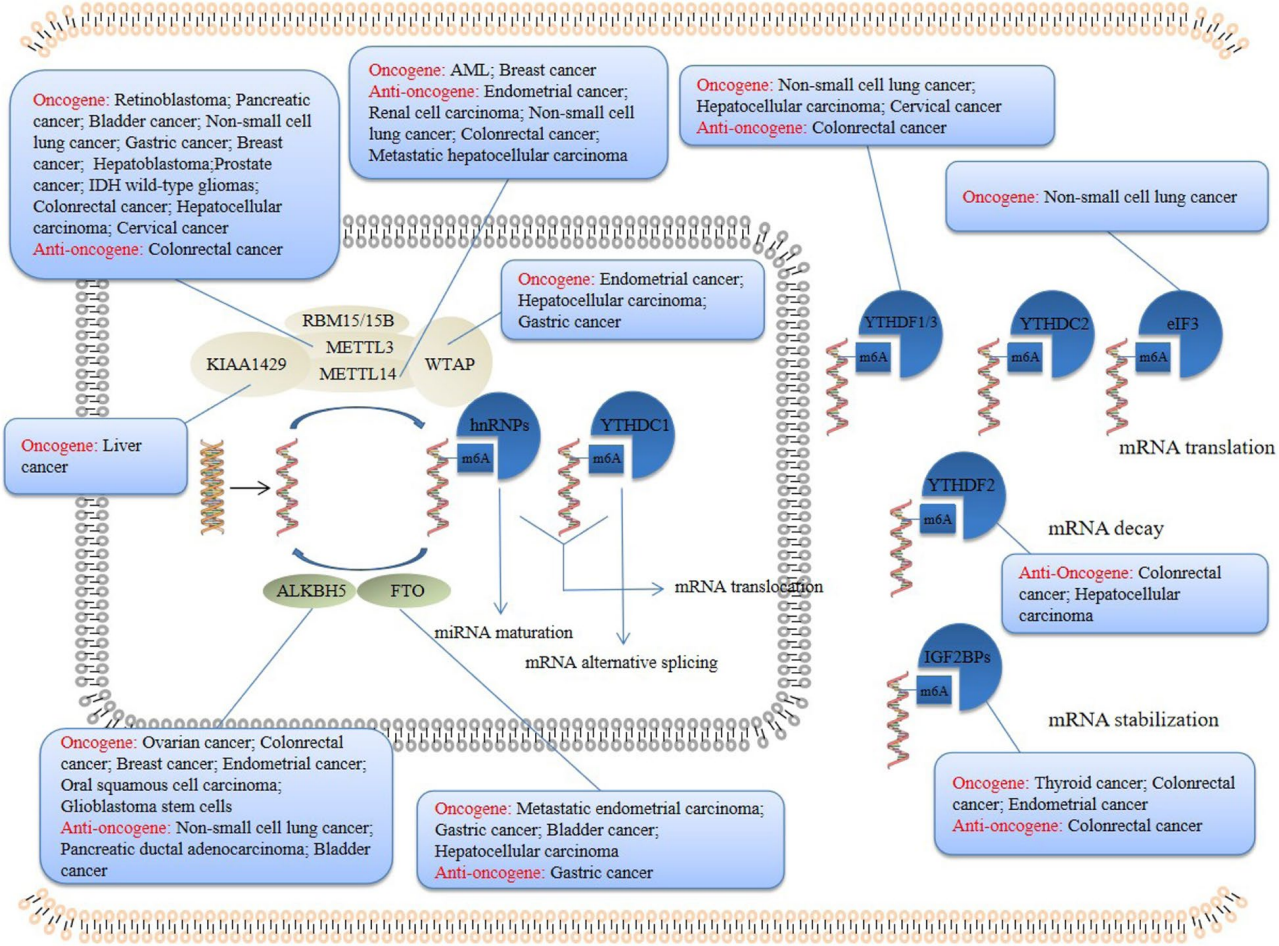
The function, expression location and function in different tumors of m6A methylases, demethylases and recognition proteins

As a central signaling pathway controlling tumor metabolism, mTOR signaling pathway is one of the signaling pathways deeply studied in tumor. The abnormal activation of mTOR signaling pathway is considered to be one of the key signaling pathways regulating tumor metabolism. MTOR is a serine / threonine kinase. There are mTORC1 and mTORC2 complexes in cells, which are highly conserved in evolution. Because its C-terminal is homologous with the catalytic domain of phosphatidylinositol kinase (PI3K), it belongs to the PI3K-related protein kinase family. In hematological malignancies, over-activated mTOR is associated with other metabolic regulators, such as AMPK and HIF-1α, in combination with microenvironment stimulation, which can regulate the activities of glycolytic enzymes in a directly or indirectly way to obtain a new glycolytic phenotype [31]. However, recent studies have found that mTOR signaling pathway may be closely related to the methylation of m6A in regulating tumor metabolism. In gastric cancer, inhibition the level of m6A methylation can activate Wnt and PI3K-Akt-mTOR signaling pathways, thus promote the progression of gastric cancer. Conversely, promote the expression of m6A methylation reverses these molecular changes in gastric cancer, suggesting that the carcinogenic phenotype and activation signaling may be directly regulated by m6A-related enzymes, rather than directly controlled by the target genes regulated by m6A methylation [32]. Meanwhile, METTL3 can directly activate PI3K-Akt-mTOR signaling pathway and promote the proliferation, migration and invasion of cancer cells in retinoblastoma [33] (Fig. 1).

**Fig. 2:**
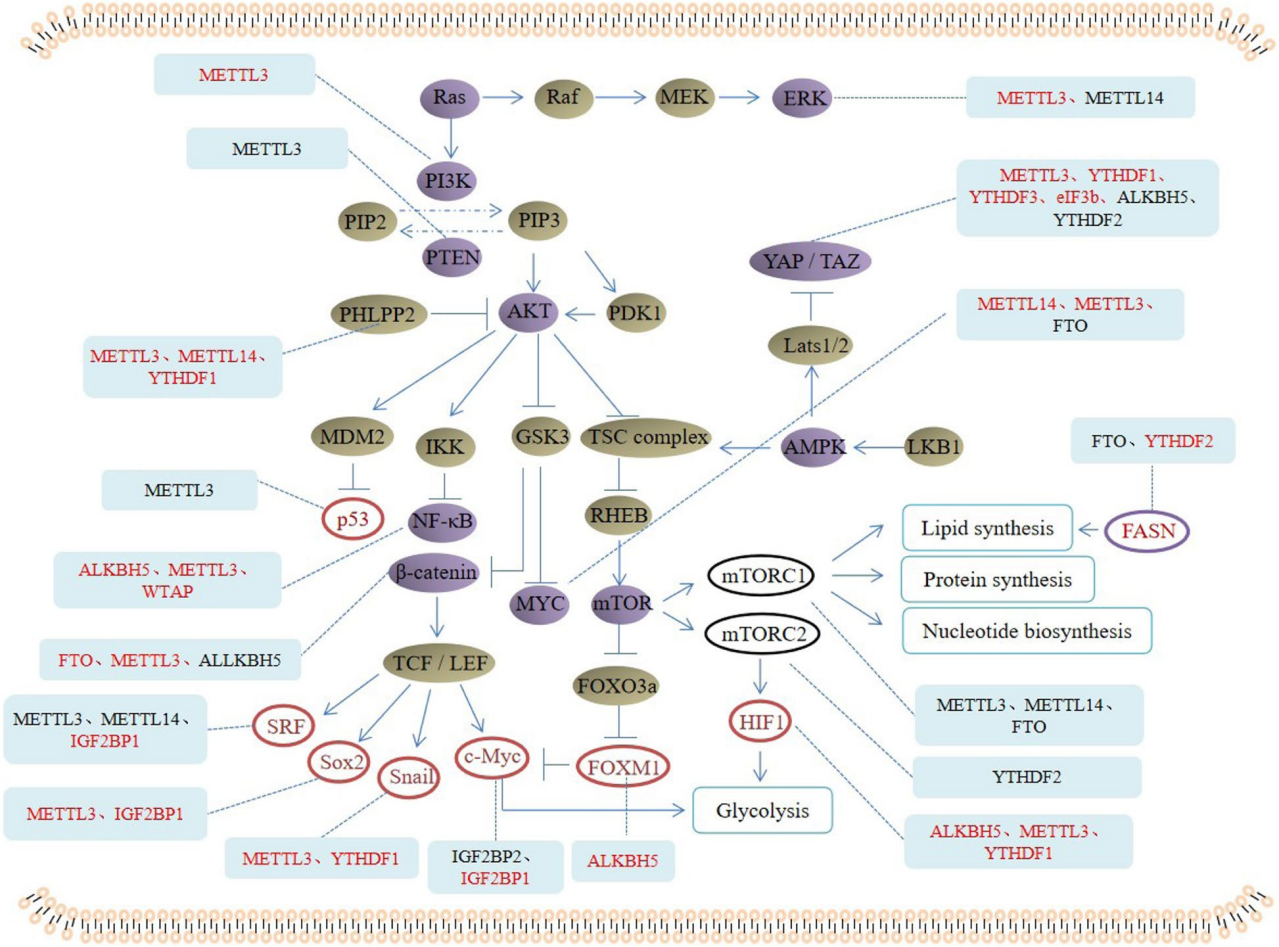
M6A methylases, demethylases and recognition proteins regulate signaling pathways, transcription factors and metabolic enzymes related to cancer metabolism. The red font indicates that there is a positive regulation between m6A methylases and proteins, and the black font indicates that there is a negative regulation between m6A methylases and proteins

Binding the ligand with membrane receptor can trigger the activation of p85 and recruit p110, and then catalyze PIP2 on the inner surface of the membrane to generate PI3P, which further activate Akt and PDK1. Akt can directly phosphorylate PRAS40, and activates mTORC1 pathway, therefore the downstream activated S6K1 can phosphorylate ribosomal 40S protein S6, initiate the translation of 5’-end of mRNA and stimulate the ribosomal protein synthesis. In addition, mTORC2 complex including PRR5, PRR5L and mTOR components, and downstream effectors are a series of cytoskeleton regulators, which can directly activate SGK1 and PKC-α, further regulate ion transport, cytoskeleton morphology and cell growth. It is found that the decreasing of m6A methylation in 70% endometrial cancer patients may be due to the mutation of METTL14 or the down-regulated expression of METTL3 (Supplementary Table 1). Decreasing methylation of m6A leads to decreasing translation of negative regulator of Akt, PHLPP2, and increasing expression of the positive regulator of Akt, mTORC2, thus promotes the proliferation of endometrial cancer cells. The difference is closely related to the specificity of m6A readers for transcription. It is found that PHLPP2 is the target of YTHDF1, and YTHDF1 promotes the translation of m6A methylated transcript, while mTORC2 is the target of YTHDF2, and YTHDF2 promotes the decay of m6A methylated transcription [21, 34, 35] (Fig. 2). Omeprazole pretreatment can enhance the inhibitory effects of chemotherapeutic drugs such as 5-FU, DDP and TAX on gastric cancer cells. This is because omeprazole induced FTO inhibition increases the expression of m6A methylation level, enhances the activation of mTORC1 signaling pathway, inhibits the transcription of apoptosis-related gene DDIT3 and autophagy, thus improves the anti-tumor effect of chemotherapeutic drugs on gastric cancer cells [36].

### The m6A and MAPK

Mitogen activated protein kinase (MAPK) signaling pathway is a critical area to induce tumor, which is involved in a series of cellular physiological activities. MAPK is mainly composed of four subfamilies: extracellular signal regulated kinase (ERK), p38 mitogen activated protein kinase (p38 MAPK), c-Jun N-terminal kinase (JNK) and extracellular signal regulated kinase 5 (ERK5). Among them, RAS-Raf-MEK-ERK signaling pathway has been widely studied and found to be inextricably linked with tumor. Researches show that MAPK signaling participates and plays an essential role in glycolysis. It is reported that as for p38 MAPK participates in aerobic glycolysis through regulating PFKBF3 and Glut2 [37]. Activation of MAPK/Erk signaling can induce c-Myc transcription, thus promoting glycolysis [38].

In pancreatic cancer, METTL3 deficient cancer cells are highly sensitive to gemcitabine, 5-fluorouracil, cisplatin and radiotherapy [39]. This is because METTL3 can regulate the cascade of MAPK, leading to resistance to chemotherapy and radiotherapy [39]. In renal cell carcinoma, ATP receptor P2RX6 may regulate Ca^2+^ mediated p-ERK1/2-MMP9 signaling pathway, and increase the invasion ability of RCC cells. METTL14 can increase P2RX6 precursor mRNA splicing, decrease P2RX6 protein translation and decrease P2RX6 mRNA and protein levels through m6A methylation [40]. METTL3 can promote activation of MAPK by promoting the methylation of pri-miR-1246 and targeting SPRED2 in colorectal cancer. Down-regulating SPRED2 further reverses the inhibition of MAPK pathway and promotes the invasion and metastasis of tumor cells [41].

### The m6A and PTEN

PTEN was discovered as a tumor suppressor at first, which mainly through negatively regulating PI3K-Akt signaling pathway. Thus, PTEN can also participate in tumor progression and glucose-lipid metabolism. PI3K can phosphorylate the metabolite PIP2 to PIP3, while the lipid phosphatase PTEN can dephosphorylate PIP3 and change it back to PIP2, thus inhibiting its accumulation in cells and terminating the PI3K signaling pathway. In bladder cancer, METTL3 interacts with DGCR8, and actively promotes the maturation of pri-miR-221/222 in a m6A dependent manner, further reduces the expression of PTEN and promotes the proliferation of bladder cancer [42]. As we all known, hepatitis B virus is a direct factor leading to liver cancer. Studies show that hepatitis B virus can significantly increase the m6A modification of PTEN, leading to the instability of PTEN expression, and reduce the expression of PTEN. The expression of PTEN can directly increase the activation of IRF-3, promote the nuclear transcription of IRF-3, thus affecting the synthesis of interferons, and influence the occurrence and development of liver cancer [43].

### The m6A and AMPK

AMPK, the signaling pathway which looks very similar to MAPK, with the full name is AMP-activated protein kinase. It can regulate energy homeostasis, and involved in a variety of signal transduction pathways. It is one of the central regulators of eukaryotic cells and maintains the smooth operation of cell physiological activities. When the expression of AMP and ADP increases, AMPK is activated. The activated AMPK pathway can affect metabolism recombination by phosphorylating the substrates and transcriptional regulators. In fact, AMPK and mTOR signaling pathway are also closely related. In the case of nutritional deficiency, AMPK can directly phosphorylate Raptor, block the ability of mTORC1 kinase complex to phosphorylate its substrate, and inhibit cell growth. In breast cancer cells, low-dose SFN can play an anti-tumor role by reducing ATP and AMPK activation pool and stimulating energy stress induced by autophagy, which is caused by promoting DNA hypomethylation and reducing the methylation level of m6A, promoting genetic instability of cancer cells and inhibiting tumor progression [44].

### The m6A and Wnt

The function of Wnt signaling pathway is mostly in the regulation of embryonic development and cancer. Wnt-β-catenin pathway can lead the accumulation of β-catenin in tumor cytoplasm and promote translocation of transcription coactivator/LEF family to the nucleus. Abnormal Wnt signaling is considered to be the driving factor of metabolic changes in glycolysis, glutamine decomposition and adipogenesis, which is crucial for the survival of cancer stem cell population [45]. Glutamine deficiency in cancer stem cells can reduce Wnt signaling activity and induce the increasing phosphorylation of β-catenin. Therefore, the regulation of glutamine on stem cell-like cancer cells is partly through the phosphorylation and degradation of β-catenin mediated by reactive oxygen species [46].

In hepatoblastoma, the abnormal expression of METTL3 can promote tumor progression, and the modification of m6A in tumor cells generally increases. CTNNB1, as a key component of Wnt signaling, its m6A abundance increases significantly with the expression of METTL3, which enhances the stability of CTNNB1 and then activating Wnt-β-Catenin signaling pathway [47]. However, in gastric cancer cells, inhibition of m6A methylation can activate Wnt signaling pathway to promote tumor cell proliferation and invasion, while FTO knockout can reverse these molecular and behaviors changes [32]. These results show that distinct m6A methylation status of Wnt-β-catenin signaling might represent opposite role in different tumors. In metastatic endometrial carcinoma, FTO can catalyze demethylation 3’-UTR region of HOXB13 mRNA, eliminate the recognition effect of YTHDF2 on the m6A methylation, reduce the attenuation effect of HOXB13 mRNA, and increasing the expression of HOXB13 can activate Wnt signaling pathway, thereby promoting tumor invasion and metastasis [48]. In pancreatic ductal adenocarcinoma, silencing ALKBH5 can promote malignant biological behaviors of cancer cells, and also promote the drug resistance of cancer cells to chemotherapy. ALKBH5 can increase the mRNA expression of Wnt inhibitor factor 1 (WIF-1) by reducing the m6A modification of WIF-1 3’-UTR, and then regulates Wnt-β-catenin signaling. Recovery experiment shows that the up-regulation of WIF-1 by ALKBH5 can be restored by methylation inhibitor DAA [49].

### The m6A and Hedgehog

Hedgehog signaling molecule is a kind of local protein ligand secreted by signal cells, which has a small range of action and its production is strictly controlled by time and space. Hedgehog signaling pathway is responsible for controlling cell fate, proliferation and differentiation of cells. Studies demonstrate that when aberrant activating the pathway can cause the occurrence and progression of tumor, which through regulating glycolysis and glutaminolysis [50, 51]. However, there are limited reports on the regulation of Hedgehog pathway by m6A methylation in tumors. In prostate cancer cells, silencing METTL3 reduces the m6A modification of Gli1, an important component of Hedgehog pathway, and reduces the expression of Gli1, as well as the expression of downstream of hedgehog pathway, which promotes tumor cell apoptosis [52].

### The m6A and NF-κB

NF-κB is one of the well-known tumor-related signaling pathways, which is often shows in form of homodimer or heterodimer, with p65 and p50. However, NF-κB can be inactivated in cytoplasm due to the formation of trimer complex by binding with IkB protein. When the upstream factor TNF binds to the receptor on the membrane surface, the receptor conformation changes and transmits the signal to IKK kinase, which phosphorylates IkB protein and dissociates it from the trimer. Then NF-κB dimer rapidly enters the nucleus from the cytoplasm and promotes the transcription of downstream-related genes, such as CyclinD1, c-Myc, VEGF and so on. Researches show that NF-κB signaling pathway not only participates in glycolysis, but also regulates fatty acid synthesis [53, 54].

When co-cultured M2 macrophages with ovarian cancer cells, high expression of TLR4 activates NF-κB and up-regulates the expression of ALKBH5, decreases the m6A methylation level of NANOG thus promotes the expression of NANOG in ovarian cancer, thus promoting the occurrence and progression of ovarian cancer [55]. This might be due to NANOG promotes the occurrence and stemness of ovarian cancer via glycolytic enzyme HK2 in ovarian cancer [56]. METTL3 is significantly up-regulated and mediates AFF4-NF-κB-MYC signaling pathway, thus promoting the malignant biological behaviors of bladder cancer cells [57]. In addition, WTAP is overexpressed in endometrial carcinoma, which promotes the proliferation, migration and invasion of cancer cells. WTAP knockout significantly increases the expression of CAV-1, causing the decreases of NF-κB signaling significantly due to it acts as the downstream of CAV-1. WTAP can methylate the 3’-UTR of CAV-1 and down-regulate the expression of CAV-1 to activate NF-κB signaling pathway, thus promoting EC progression [58].

Many studies show that NF-κB signaling pathway is involved in regulation tumor cell immune microenvironment. However, the researches on the role of m6A modification in the regulation of immune microenvironment are very limited. Yin et al. found that METTL3 deficient mice showed the increase of tumor infiltration by M1 or M2-like tumor-associated macrophages and regulatory T cells, and weakened the efficacy of PD-1 checkpoint blockers. The METTL3 deficient mice decreases m6A methylation level of SPRED2, which can’t be recognized by YTHDF1, thus the reduction of SPRED2 translation leads to enhance NF-κB activation and promote tumor progression [59].

## The m6A and transcription factors

Transcription factors are proteins that regulate gene transcription process. It is an important part in regulating signal pathways, also known as the third messenger. Therefore, comprehensive analysis of the important role of transcription factors in tumor metabolism is a powerful supplement to investigate the role of signaling pathways in tumor metabolic recombination. Therefore, it is very necessary to find out the common signaling pathways and transcription factors correlated with metabolic recombination of tumor cells, immune cells and fibroblasts in the tumor microenvironment, so as to understanding the impacts of metabolic recombination on tumor microenvironment as a whole and promote tumor progression.

### The m6A and HIF-1

HIF-1 is a well-known hypoxia inducible factor, hypoxia and tumor are mutual positive feedback. Firstly, the increasing oxygen consumption caused by the proliferation of tumor cells promotes the hypoxia environment in the tumor microenvironment. On the contrary, the hypoxia microenvironment can promote the proliferation, differentiation, energy metabolism and drug resistance of tumor cells through various factors and signaling pathways, thus forming a positive feedback loop to promote the malignant progress of tumor. It is one of the important reasons for poor prognosis of cancer patients. HIF-1α and HIF-2α are heterodimeric transcriptional activators, which consist of HIF-1α and HIF-2α subunits regulated by O_2_ and HIF-1β subunits constitutively expressed [60]. Due to the role of HIF plays in tumor microenvironment regulation, HIF can widely participate in tumor metabolic reorganization, including glucose metabolism, lipid metabolism and so on. HIF shows an essential role in regulating glycolysis in the development of various cancers, including regulates glycolysis enzymes of HK2, GLUT1, PKM2 and et al. [61, 62]. Studies have shown that a variety of m6A methylases can participate in the regulation of HIF-1 methylation level and expression, and then participate in tumor progression.

In breast cancer, hypoxia induces demethylation of m6A, mainly HIF-1α and HIF-2α, which is highly expressed under hypoxia, can activate the gene transcription of ALKBH5, reduce NANOG m6A methylation and stabilize its stability, increase its protein expression, and promote the phenotype of breast cancer stem cells [63] (Supplementary Table 2). It is well known that the activation of autophagy is one of the important ways for cancer cells to survive in hypoxia. In hepatocellular carcinoma, HIF-1α promotes the expression of YTHDF1 by binding to the promoter of YTHDF1, and then combines with ATG2A and ATG14 by m6A modification to promote their translation and malignant progression of cancer [64].

In HCC, the expression of HBXIP (hepatitis B virus X-interacting protein) is up-regulated, which positively regulates the expression of downstream methylase METTL3 and promotes HIF-1α expression, sustain high level of glycolysis, thus promote malignant biological behavior of HCC cells [65]. In endometrial carcinoma, hypoxia and high level of ALKBH5 expression promote the transcription of SOX2 through demethylation, thereby increasing the stem cell-like phenotype of endometrial carcinoma. Knockout HIFs or ALKBH5 can significantly reduce its tumor initiation ability [66].

### The m6A and FOXM1

FoxM1 is a common transcription factor regulating tumor cells, which is mainly involved in the regulation of cell cycle and is closely related to the abnormal proliferation and division of tumor cells. In fact, FoxM1 has a strong regulatory effect on the metabolic recombination of tumor cells. Inhibition of FoxM1 expression significantly decreases the activation and expression of GLUT1 and HK2, and then inhibits the aerobic glycolysis and cell proliferation [67]. In gastric cancer, FoxM1 targets transcription and activation of Prx3, promotes stemness and metabolic reprogramming of gastric cancer cells, and increases the expression of mitochondrial fatty acid oxidative phosphorylation rather than glycolysis, which promotes drug resistance of tumor cells [68]. These studies suggest that FoxM1, as a transcription factor, can target metabolism-related genes and regulate tumor cell progression.

It is found that ALKBH5 is closely related to the expression of FOXM1, regulating the level of FOXM1 methylation and tumor progression in many tumors. In glioblastoma stem cells, m6A demethylase ALKBH5 is highly expressed, which directly targets the new transcripts of FoxM1 through demethylation, thus increasing the expression of FoxM1, and promoting the stemness and proliferation of tumor cells. HuR, as an RNA binding protein, promotes the expression of FoxM1 by binding to unmethylated 3’-UTR, and participates in the regulation of FoxM1 by ALKBH5. However, it is worth noting that FOXM1-AS is a long non-coding RNA of antisense FoxM1, which can promote the interaction between ALKBH5 and FoxM1 new transcripts [69]. In oral squamous cell carcinoma, DDX3 can directly regulate the expression of ALKBH5, reduce m6A methylation in FoxM1 and NANOG new transcripts, lead to chemotherapy resistance [70].

### The m6A and P53

At first, p53 was discovered as an oncoprotein antigen. Later, it was thought that p53 is an important oncogene. In fact, p53 is a broad-spectrum tumor suppressor gene. Wild-type p53 can promote cancer cell apoptosis, and its inactivation plays an important role in tumor formation. In malignant tumors, more than 50% of patients will have p53 mutations. Functionally, p53 acts as a transcription factor which can regulate cell cycle initiation. However, the function of p53 is not limited to directly regulation of cell cycle and apoptosis, which also widely involved in the metabolic reorganization of tumor cells through a variety of signaling pathways. In colorectal cancer, wild-type p53 targets miR-143-3p-PDK2 signaling pathway to regulate tumor cell glucose metabolism and influence chemoresistance [71]. In addition, p53 inhibits glucose consumption and NADPH expression by binding with G6PD [72]. Compared to glycolysis, wild-type p53 prefers to promote mitochondrial respiration when regulates cell metabolic diversity, which partly through trans-activation of SCO2, the molecule participates in oxidative phosphorylation [73]. Contrary to the function of wild-type p53, mutant p53 in tumor cells is conducive to aerobic glycolysis. Mutant p53 can enhance the transport of glucose transporter GLUT1 to plasma membrane, thus increasing the input of glucose [74]. In addition, p53 can directly participate into the trans-activation of GLS2, and GLS2 can mediate glutamine as the energy source of mitochondria [75].

However, the studies focus on investigate m6A modification of p53 in regulating cancer metabolism is very limited. As we all known, mutant p53 protein promotes the growth and chemoresistance of cancer cells. Silencing METTL3 can inhibit the formation of p53 pre-mRNA, significantly increase phosphorylated p53 protein (Ser15) and play its role in heterozygous R273H mutant cells, inhibit the expression of p53 R273H mutant protein, and restore wild-type p53 protein expression. Therefore, R273H cells are sensitive to anti-cancer drugs [76]. Arsenic is a non-metallic element with strong toxic effect and can cause cancer through epigenetics. When human keratinocytes are exposed to arsenite, increases the expression of METTL3, which inhibits the activation of p53 and reduces the phosphorylation and acetylation of p53, mediates the transformation of human keratinocytes induced by arsenite [77].

### The m6A and YAP

YAP is an intracellular connexin and transcriptional co-activator, which is the downstream of Hippo signaling pathway. YAP and TAZ are carcinogenic drivers of human solid tumors, and they can interact with transcription factors to participate in the occurrence and development of tumors [78]. Inhibition of glycometabolism or glycolysis results in the decrease of Yap / TAZ transcriptional activity [79].

In non-small cell lung cancer, METTL3 can recruit YTHDF1/3 and eIF3b to form translation initiation complex, thus promoting the translation of YAP. In addition, METTL3-YTHDF3 complex can improve the stability of MALAT1, and promote the invasion, metastasis and chemoresistance of lung cancer cells through YAP mediated miR-1914-3p [80]. In colorectal cancer, LncRNA GAS5 interacts with the WW structure of YAP, promotes its phosphorylation and ubiquitin mediated degradation, and promotes the transfer of endogenous YAP from nucleus to cytoplasm. As the downstream of YAP, m6A reader YTHDF3 can reversibly and selectively bind and promote the degradation of m6A modified GAS5, and the up-regulation can reverse the inhibition of GAS5 mediated by YTHDF3. Thus, negatively feedback loop of GAS5-YAP-YTHDF3 is formed to inhibit the progression of colorectal cancer [81]. In non-small cell lung cancer, ALKBH5 can reduce the m6A modification of YAP, reduce the expression of YAP mediated by YTHDFs, and YTHDF family can competitively bind to YAP for regulation. YTHDF1 promotes the translation of YAP mRNA through interaction with eIF3a, YTHDF2 promotes the degradation of YAP mRNA through AGO2 system, and YTHDF3 binds YAP precursor mRNA. In addition, ALKBH5 can inhibit the miR-107-LATS2 signaling axis, reduce the activity of YAP and inhibit the growth and metastasis of tumor cells, which may be a potential strategy for the treatment of lung cancer by targeting ALKBH5 [82].

### The m6A and c-Myc

MYC, also named c-Myc, is the most important transcription factor and extensive nuclear oncogene at present. As an important transcription factor, the most famous function of c-Myc is to cooperate with other three transcription factors Sox2, Oct4 and KLF4 to reprogram fibroblast cells into pluripotent stem cells. In colorectal cancer, GLCC1 stabilizes transcription factor c-Myc by regulating the ubiquitination of c-Myc, which further regulating the transcriptional modification of downstream genes, promoting proliferation and survival of cancer cells by enhancing glycolysis [83]. The studies showed that c-Myc plays an important role in regulating cancer metabolism.

As an independent prognostic factor, c-Myc knockout can inhibit the expression of YTHDF1, thereby inhibiting the proliferation and chemoresistance of tumor cells [84]. In lung cancer, miR-338-5p can inhibit the expression of METTL3, thereby decreasing the m6A modification of c-Myc, down-regulating its expression and inhibiting the proliferation, invasion and migration of lung cancer cells [85]. In OSCC, METTL3 targets the 3’-UTR of c-Myc transcript to increase the m6A modification. YTHDF1 cooperates with the METTL3 m6A effects to enhance the stability of c-Myc, therefore knockout METTL3 can inhibit the malignant progression of tumor cells [86]. In gastric cancer, HDAC3 can accelerate the invasion and migration of gastric cancer cells by targeting FOXA2. FOXA2 can bind to the promoter of m6A eraser FTO and reduce its expression. However, FTO can reduce the methylation of c-Myc in gastric cancer cells and stabilize its expression, thereby affecting the tumor initiation activity of gastric cancer cells [87]. In addition, the expression of METTL3 in gastric cancer cells can also affect the carcinogenic function of tumor cells, and its overexpression can promote tumor progression. MCM5 and MCM6 are the molecules targeted by c-Myc, and METTL3 can regulate MCM5 and MCM6 through m6A modification. Knockout METTL3 significantly reduces the m6A levels of MCM5 and MCM6, thus inhibiting carcinogenic of gastric cancer [48]. In the acute myeloid leukemia (AML) carrying t (11q23), t (15;17) or t (8;21), METTL14 is up-regulated, and the down-regulation of METTL14 accompany with myeloid differentiation. Silencing METTL14 can promote the terminal differentiation of AML cells, thus inhibit proliferation of AML cells. METTL14 plays a carcinogenic role by modifying the downstream expression of MYB-MYC in an m6A-dependent manner. Inhibition METTL14 expression results in a significant decreasing of the half-life of MYB and MYC transcripts, thus inhibiting tumor progression [88].

### The m6A and SRF

SRF (serum response factor) is a member of MADS box transcription factor superfamily. Its expression is highly conserved and participates in many important life activities of cells. Although the research on the relationship between SRF and metabolism reprogramming is very limited, some studies have shown that SRF is involved in high glucose induced epithelial mesenchymal transition or high glucose induced damage to retinal ganglion cells. High glucose stimulates the overexpression of SRF, which promotes the epithelial mesenchymal transition of human peritoneal mesothelial cells induced by high glucose through directly binding to Snail promoter [89]. In tumor cells, SRF can promote cell reprogramming into multifunctional cells [90]. SRF, together with MRTF and TCF, can regulate the migration, invasion, growth and proliferation of tumor cells in a signal and cytoskeleton dependent manner [91]. The synergistic effect of Myc and RhoA-SRF pathway has a synthetic lethal effect, which is caused by the insufficient utilization of glutamine, suggesting that Myc and RhoA-SRF have metabolic coordination in maintaining the vitality of cancer cells [92].

IGF2BP1 promotes SRF expression in an m6A dependent manner, enhances SRF dependent transcriptional activity at the post-transcriptional level, and promotes tumor cell proliferation and invasion. The results show that knockout METTL3 and METTL14 down-regulates the expression of SRF in cells, while in the SRF-3’-UTR mutant cancer cells, the deletion of METTL3 and METTL14 don’t affect the expression of SRF. These results suggest that IGF2BP1 enhances SRF expression through a conserved 3’-UTR and m6A dependent manner, and then regulates tumor progression [93]. However, the researches on regulation of SRF by m6A methylation in tumor are very limited, more researches are needed to clarify the role of m6A methylation in regulating SRF in tumor metabolism.

### The m6A and OCT4

OCT4 is a member of the POU transcription factor family, which has many subtypes. The translated proteins contain a conservative DNA binding domain, namely the POU binding domain, which is involved in regulating the stem cell stemness and differentiation. In human embryonic stem cells, silencing GLUT3 leads to the decrease of glucose uptake, lactate production and OCT4 expression, suggesting that the self-renewal of human embryonic stem cells is regulated by glucose uptake rate. The glycolysis enzyme PKM2 increases in the hypoxia condition, and silent PKM2 reduces the expression of OCT4 [94]. PKM2 translocation can help cancer cells survive under metabolic stress. The combination of PKM2 and OCT4 promotes the expression of tumor stem cell-related genes, which may enrich tumor stem cell group under metabolic stress [95]. In hepatoma cells, knockout of YTHDF2 results in decreasing the stemness of cancer cells. This is due to knockout of YTHDF2 can reduce the m6A level of OCT4 5’-UTR, and the expression of OCT4 protein, thereby affecting the stemness of tumor cells [96].

### The m6A and SOX2

Sox2 is one of the members of Y-related HMG protein family in Sox region. It is an essential factor for embryonic development and the necessary condition for maintaining the self-renewal of embryonic stem cells. In addition, Sox2 can act as one of the initial factor of inducing pluripotent stem cells. In ovarian cancer, as a transcription factor, Sox2 directly binds to the promoter of ST6Gal-I to drive transcription and increases in N-glycosylated protein α2-6 linked sialic acid, promotes ST6Gal-I and α2-6-linked sialic acid expression, regulates glucose metabolism in cancer cells [97].

In endometrial cancer, PADI2 (peptide arginine deaminase II) can convert arginine residues to citrulline, participates in the regulation of amino acid metabolism, catalyzes the citrullination of MEK1-R113/189, promotes the phosphorylation of ERK1/2, and activates the expression of IGF2BP1. IGF2BP1 can bind to the m6A site of Sox2 3’-UTR to inhibit its degradation, thus promoting the progression of endometrial cancer [98]. Studies have found that METTL3 plays a role of proto-oncogene in colorectal cancer, promotes the methylation level of Sox2 in a m6A dependent manner, prevents Sox2 degradation through the synergistic effect of IGF2BP2, and maintains the expression of Sox2, thus promoting the progression of colorectal cancer [99]. In bladder cancer, the over-expression of METTL3 increases m6A methylation status, regulates the expression of AFF4, promotes its transcription by combining with the promoter region of Sox2, and promotes the self-renewal of cancer stem cells [100].

### The m6A and ETS-1

ETS-1, a member of ETS transcription factor family, has a conserved ETS-DNA binding domain and is a key factor in NK cell differentiation. In pancreatic cancer cells, silencing ETS-1 reduces the expression of GLUT-1, interferes with glycolysis and reduces glucose utilization and lactate production, reduces the energy produced in the form of ATP, and inhibits the vitality and invasion ability of tumor cells [101]. WTAP is an important part of m6A writer, and its main function is to recruit methylase METTL3 and METTL14. As a transcription activator, ETS1 is regulated by Ras-RAF-MEK-ERK signaling pathway [102]. In HCC, WTAP interacts with RNA binding protein HuR to regulate the transcription inhibition of ETS1 by m6A modification, and then regulates the downstream p21-p27 signal axis to regulate the progression of HCC [103].

### The m6A and Snail

Zinc finger transcription factor superfamily Snail family is very conservative in evolution. The amino acid end of the Snail family members contains evolutionarily conserved SNAG domain, which plays an important role in the regulation of transcription inhibition and participates in embryonic development and tumorigenesis. Snail itself is a highly unstable protein, regulated by comprehensive and complex network signals. In gastric cancer, up-regulation of Snail promotes lactate production and glucose utilization, reduces the expression of FBP1, which is the rate limiting enzyme of gluconeogenesis, plays a positive role in regulating glucose metabolism, promotes glycolysis, and then promotes EMT of tumor cells [104].

In HCC, knockout of METTL3 decreases the expression of Snail and inhibits the progression of HCC. Sumo binding enzyme UBC9 regulates the SUMOylation of METTL3, controls homeostasis and promotes the accumulation of Snail, thus promotes the progression of HCC [105]. On the contrary, it is found that the expression of Snail mRNA decreases in the cells with SUMO mutant of METTL3 [106]. In addition, m6A can trigger the polysome mediated translation of Snail, which is in the CDS region of Snail instead of the 3’-UTR region, promote Snail transcription and participate in regulation EMT phenotype of tumor cells. Meanwhile, m6A reader YTHDF1 synergistically increases the translation of Snail mRNA which mediated by m6A methylation [107].

## The m6A and non-coding RNA

Non-coding RNA is a kind of non-coding transcripts that do not encode proteins, but can produce non-coding transcripts that regulate gene expression and protein function, which plays a role in regulating development, differentiation and metabolism in terms of physiological and pathological processes. Studies have revealed that about 90% of the genes in the eukaryotic genome are transcriptional genes. However, only 1–2% of these transcriptional genes encode proteins, and most of them are transcribed as ncRNA. Studies have shown that non-coding RNA not only plays a role at the transcriptional and post transcriptional levels, but also participate in the epigenetic regulation of gene expression. Therefore, exploring the regulatory role of m6A on non-coding RNA has attracted extensive attention. As an important part of genes regulating tumor progression, the research of non-coding RNA in tumor metabolism is relatively novel. Mining the role of non-coding RNA in tumor metabolism is of great potential. A large number of studies show that non-coding RNA is of great significance in the regulation of tumor metabolism.

### The m6A and miRNA in cancer metabolism

MiRNA is a non-coding single stranded RNA molecule with a length of about 22 nucleotides encoded by endogenous genes, which participates in regulation of post-transcriptional gene expression. Large number of studies shown that miRNA is involved in the regulation of tumor cells metabolism. However, the relevant researches of m6A methylation play in miRNAs in regulating tumor cells metabolism is ongoing. The regulation of miR-221 and miR-222 is often synchronized, because they are located very close in human nucleic acid sequence, with the same "seed sequence". MiR-221/222 cooperatively participates in insulin resistance, leading to metabolic syndrome and aggravating the disorder of glucose and lipid metabolism, which might participate in chemotherapy resistance of cancer [108]. METTL3 is significantly increased in bladder cancer, which can interaction with DGCR8 promotes the maturation of pri-miR-221/222 family in an m6A dependent manner, reduces the expression of downstream gene PTEN through synergistic effect, and promotes the progression of bladder cancer and poor prognosis of patient [42] (Supplementary Table 3). MiR-375 is highly expressed in islets β cells, and involved in regulation β cell proliferation and differentiation. During glycolysis, overexpression of miR-375 in islet cells increases the expression of PDK4 and decreases the expression of pyruvate carboxylase [109]. METTL14 regulates the expression of miR-375 through DGCR8, and then inhibits the growth of cancer cells by targeting downstream YAP1 pathway, and also inhibits the invasion and migration of colorectal cancer cells [110]. Long non-coding RNA XIST regulated by m6A methylation can target miR-126 and regulate glucose metabolism of glioma cells, thereby affecting tumor cell proliferation [111]. Down-regulation of METTL14 is an adverse factor for recurrence-free survival and prognosis of patients with hepatocellular carcinoma. By interacting with DGCR8, METTL14 positively regulates the expression of pre-miR-126, and miR-126 also reversely inhibits the inhibitory effect of METTL14 on tumor cell metastasis [112].

### The m6A and lncRNA in cancer metabolism

LncRNA is a non-coding RNA with a length of more than 200 nucleotides. Studies have shown that lncRNA plays an important role in many life activities, including epigenetic regulation, cell cycle and cell differentiation, especially cancer cell metabolism. MALAT1 is one of the long non-coding RNAs discovered in the early stage, which is highly expressed in a variety of tumors and closely related to tumor glycometabolism. MALAT1 can enhance glycolysis of tumor cells, inhibit gluconeogenesis, and promote tumor progression [113]. Combination effect of METTL3 with YTHDF3 can improve the stability of MALAT1 and increase the expression of MALAT1, thus increase the stability of YAP mRNA by regulating MALAT1-miR-1914-3p-YAP axis in non-small cell lung cancer. METTL3 can also recruit YTHDF1/3 and eIF3b from translation initiation complex, regulate the m6A modification of YAP and promote YAP translation [80]. What’s more, non-coding RNA can also regulate the expression of m6A-related methylase. In thyroid cancer, MALAT1 targets miR-204, while IGF2BP2 is confirmed to be the target of miR-204. MiR-204 up-regulates IGF2BP2 and enhances the expression of c-Myc through methylation recognition sites, which promotes the proliferation and invasion of thyroid cancer cells [114]. XIST is a long non-coding RNA that mediates the transcription silencing of genes on the X chromosome in female mammals. Study shows that XIST can regulate the glucose metabolism of glioma cells and then affect the proliferation, which is achieved by targeting miR-126 and then affecting IRS1-PI3K-Akt signaling pathway [111]. The methylation modification components RBM15 and RBM15B can recruit the methylation complex of m6A to the specific ectopic site of XIST and trigger the methylation of adjacent sites. Thus, knockout of RMB15 and RMB15B, or METTL3, can inhibit XIST mediated gene silencing. As a m6A reader, YTHDC1 can preferentially recognize m6A site on XIST, leading to the XIST mediated X-linked gene silence [115]. METTL14 knockout reduces the m6A level of downstream XIST, which can’t be recognized by YTHDF2 and inhibits its degradation, promotes the expression of XIST, and promotes the progress of colorectal cancer [116].

### The m6A and circRNA in cancer metabolism

Different from the traditional linear RNA, circRNA has a closed ring structure, which is not affected by RNA exonuclease, has more stable expression and is not easy to degrade. CircRNA binds miRNA through the competitive endogenous RNA mechanism. Recently, studies shown that circRNA is involved in the regulation of tumor cells metabolism. However, the relevant researches of m6A methylation play in circRNAs in regulating tumor cells metabolism is relative few. In glioma, Warburg effect can promote the release of circ-0072083 in exosomes of resistance glioma cells, which further promote TMZ resistance. This is due to circ-0072083 can target miR-1252-5p, thus regulating ALKBH5 to demethylate NANOG and promote its expression, which can promote TMZ resistance in glioma cancer cells [117]. In non-small cell lung cancer, circNDUFB2 can promote the interaction between TRIM25 and IGF2BPs, promote the ubiquitination and degradation of IGF2BPs, thus inhibit the proliferation and metastasis of cancer cells [118]. In HCC, due to the METTL3 and METTL14 promote the m6A methylation of circRNA-SORE, which can increase the stability of circRNA-SORE. Thus, the overexpression of circRNA-SORE can further inhibit miR-130a-2-5p and miR-660-3p, which active Wnt/β-catenin pathway and induce sorafenib resistance [119]. However, most of studies focused on the role of m6A methylation in regulating the circRNA expressions, the specific role of circRNA regulated by m6A methylation in influencing the cancer metabolism pathways need to be further investigated.

## The m6A and metabolic

### The m6A and glucose metabolism

Glucose metabolism can be divided into catabolism and anabolism. Its metabolic pathways mainly include anaerobic digestion of glucose, aerobic oxidation, pentose phosphate pathway and glycogen synthesis and decomposition. It is well known that abnormal glucose metabolism is an important part of metabolic reprogramming in tumor cells. Among them, a marker of high invasiveness of cancer cells is energy metabolism, including the increase of glycolytic activity and lactic acid fermentation, namely Warburg effect^9, 136^. Warburg effect is a typical feature of abnormal glucose metabolism in tumor. It can enhance glycolysis, glucose uptake and consumption, and then make tumor cells more easily adapt to adverse living environment for malignant proliferation. Study shows that Warburg effect can promote the release of circ-0072083 in exosomes of resistance glioma cells, which targeting miR-1252-5p to regulate ALKBH5, demethylates NANOG and promotes its expression, further promotes TMZ resistance in glioma cancer cells [117]. In non-small cell lung cancer, METTL3 induces m6A methylation of LncRNA ABHD11-AS1, enhances the stability of ABHD11-AS1 transcript to increase its expression, and promotes the proliferation of tumor cells and Warburg effect [120]. In colorectal cancer, the overexpression of m6A reader IMP2 (IGF2BP2) can stabilize ZFAS1/OLA1 axis, which increases the recruitment of OLA1, ATP hydrolysis and glycolysis, activates Warburg effect, and enhances cell proliferation and colony formation of cancer cells [121].

**Fig. 3:**
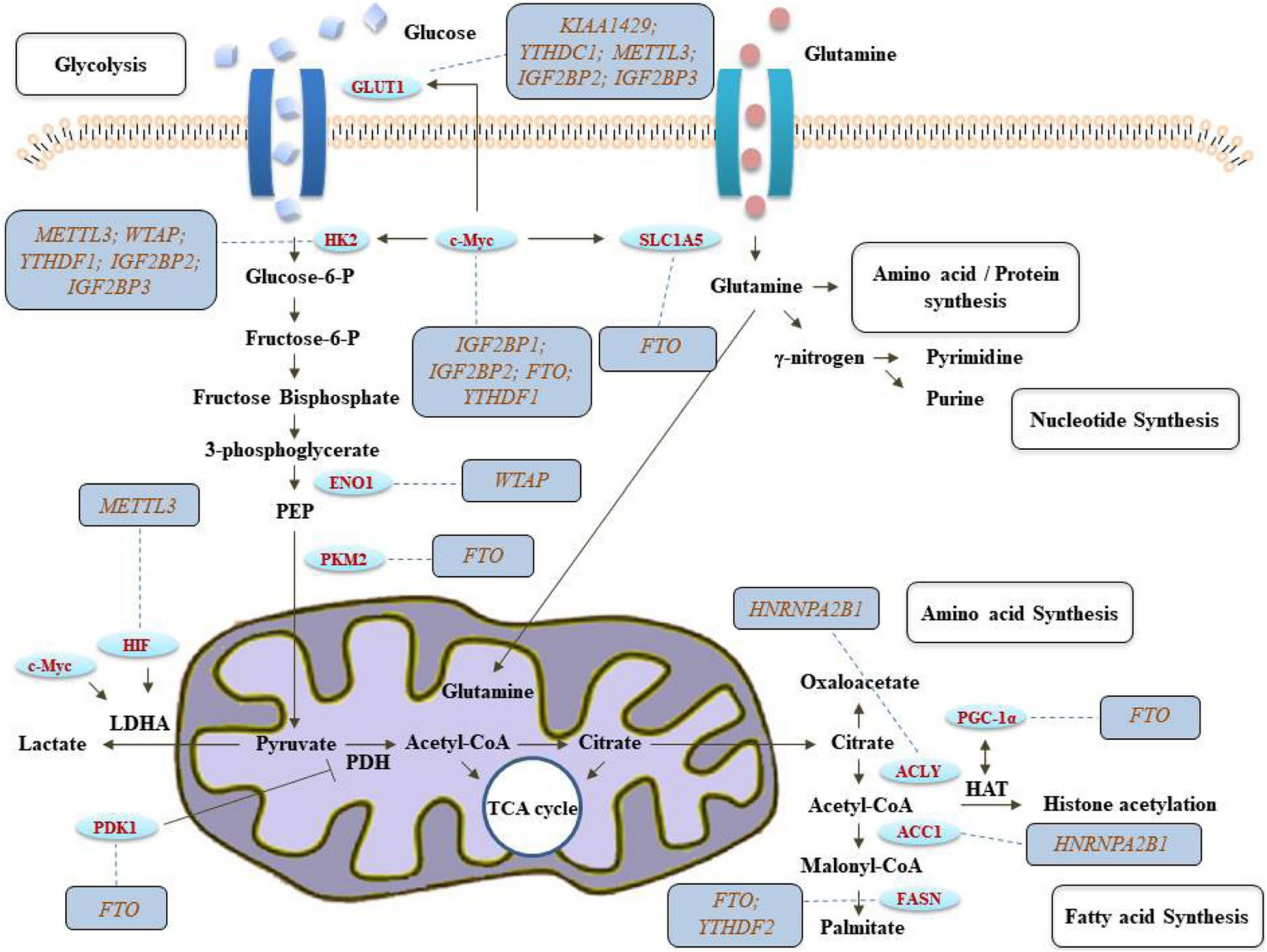
The function of m6A methylases that participate in cancer metabolism pathways, including glycolysis, amino acid synthesis, nucleotide synthesis and fatty acid synthesis. Importantly, the glycolysis of tumor cells regulated by m6A methylases mainly refers to Warburg effect, which is aerobic glycolysis

HK2 is the first important rate limiting enzyme in glycolysis (Fig. 3). METTL3 targets the 3’-UTR of HK2 mRNA. METTL3 recruits the m6A reader YTHDF1 to enhance the stability of HK2, thus promoting the Warburg effect of cervical cancer [122] (Table 2). Extracellular acidification rate (ECAR) assay shows that METTL3 can significantly promote glycolysis. After METTL3 knockout and YTHDF1 silencing, the half-life of HK2 mRNA is significantly shortened. In conclusion, the combination of METTL3 and YTHDF1 enhances the stability of HK2 [122]. In gastric cancer, m6A writer WTAP can promote the proliferation and glycolysis of tumor cells, and knockout of WTAP can inhibit tumor progression. The mechanism is that through binding the 3’-UTR of HK2, WTAP can enhance the stability of HK2 mRNA, the carcinogenic effect of WTAP and its m6A mediate regulation of Warburg effect in gastric cancer, which provides a new way and target for the treatment of gastric cancer [123].

**Table 2:** The relationship between m6A enzymes and cancer metabolism

Classification	Cancer Type	M6A Related Enzymes	Biological Behavior Changes	Related Enzymes	Official Full Name of the Enzymes	References
Glucose metabolism	Cervical cancer	METTL3; YTHDF1	Promote Warburg effect and glycolysis	HK2	Hexokinase 2	[122]
	Gastric cancer	WTAP	Promote the proliferation and glycolysis of tumor cells	HK2	Hexokinase 2	[123]
	Colorectal cancer	METTL3; IGF2BP2; IGF2BP3	Activate glycolysis pathways	HK2; SLC2A1	Hexokinase 2; Solute carrier family 2 member 1	[124]
	Gastric cancer	KIAA1429	Promote aerobic glycolysis to promote tumor progression	GLUT1	Glucose transporter 1	[126]
	Pancreatic ductal adenocarcinoma	YTHDC1	Attenuation Warburg effect to inhibit tumor progression	SLC2A1; HK1	Solute carrier family 2 member 1; Hexokinase 1	[127]
	Bladder cancer	ALKHB5	Regulate glycolysis pathways and glucose absorption, lactate and ATP production	CK2α	Casein Kinase 2α	[128]
	Hepatocellular carcinoma	FTO	Accumulate glycolysis into anabolic pathways	PKM2	Pyruvate Kinase M2	[129]
	Colorectal cancer	IGF2BP2	Promote glycolysis	c-MYC	MYC proto-oncogene	[131]
	Lung adenocarcinoma	FTO; YTHDF1	Promote tumor glycolysis and tumorigenesis	c-MYC	MYC proto-oncogene	[132]
	GBM	FTO	Promote aerobic glycolysis and promote chemoresistance	PDK1	Pyruvate dehydrogenase kinase 1	[133]
	Cervical cancer and Liver cancer	YTHDF1; IGF2BP3	Promote glycolysis and ATP generation	PDK4	Pyruvate dehydrogenase kinase 4	[134]
	Breast cancer	WTAP	Promote glycolysis and promote tumor progression	ENO1	Enolase 1	[135]
	Renal cell carcinoma	METTL14	Promote tumor cell distal lung metastasis	BPTF	Bromodomain PHD finger transcription factor	[136]
Fatty acid metabolism	Hepatocellular carcinoma	FTO; YTHDF2	Influence the content of adipogenic enzymes and intracellular lipids	FASN	Fatty acid synthase	[141]
	Esophageal cancer	HNRNPA2B1	Promote cellular lipid accumulation to promote tumor progression	ACLY; ACC1	ATP citrate lyase; Acetyl-CoA carboxylase	[143]
Amino acid metabolism	Renal clear cell carcinoma	FTO	Synthetic death with VHL and activate VEGF and PDGF	SLC1A5	Solute carrier family 1 member 5	[146]
	Colorectal cancer	IGF2BP1	Promote the tumorigenesis	MYC	MYC proto-oncogene	[147]
Mitochondrial metabolism	Renal clear cell carcinoma	METTL3	Regulate one carbon metabolism and aerobic glycolysis of tumor cells	MTHFD2; HIF-2α	Methylenetetrahydrofolate dehydrogenase 2; Hypoxia inducible factor-2α	[151]
	Renal clear cell carcinoma	FTO	Regulate mitochondrial activity and promote oxidative stress and ROS production	PGC-1α	PPARG coactivator 1α	[153]
	Breast cancer	METTL3	Inhibit apoptosis of mitochondrial, attenuate resistance to tamoxifen	AK4	Adenylate kinase 4	[154]

18F-FDG, or fluorodeoxyglucose, known as "century molecule", can accurately reflect the metabolic level of glucose, is the main imaging agent of PET-CT imaging. Due to the high metabolism of cancer cells and the increasing demand for glucose, most tumor lesions show high uptake after intravenous injection of 18F-FDG, so as to accurately determine the primary and metastatic tumor lesions in the whole body. It is found that there is a strong correlation between METTL3 and 18F-FDG uptake in colorectal cancer patients. METTL3 can directly interact with the 5’-UTR or 3’-UTR region of HK-2 and the 3’-UTR region of glucose transporter SLC2A1 (namely GLUT1), which is dependent on IGF2BP2 or IGF2BP3 to stabilize gene expression, and activate the downstream glycolysis pathway to regulate tumor cell progression [124]. Therefore, METTL3 can promote the uptake of 18F-FDG through regulating HK2 and GLUT1 in an m6A dependent manner [125]. In gastric cancer, KIAA1429 increases the m6A modification of Linc00958, which can significantly up-regulate the expression of Linc00958. In fact, Linc00958 can further increase GLUT1 stability, thus promotes aerobic glycolysis to promote tumor progression [126]. In pancreatic ductal adenocarcinoma, YTHDC1 promotes the maturation of miR-30d, and miR-30d can suppress aerobic glycolysis by binding RUNX1, regulating SLC2A1 and HK1 expression, thus attenuation Warburg effect to inhibit tumor progression [127].

In bladder cancer, the expression of ALKBH5 is down-regulated. In mechanism, ALKBH5 is linked to 3’-UTR of protein kinase CK2α, reduces CK2α in m6A dependent manner, influences the relative half-life thus affects its stability, regulates the glycolysis pathway and inhibits CK2α mediated glucose absorption, lactate and ATP production, thus inhibits the progress of bladder cells and promotes the sensitivity of cancer cells to cisplatin [128].

In HCC, the m6A methylation of PKM2 mediated by FTO can accelerate the production of translation products and promote the development of HCC. FTO knockout can induce G0/G1 phase arrest and inhibit tumor proliferation and growth in vivo [129]. Pyruvate kinase (PK) is one of the rate limiting enzymes in glycolysis. It is mainly expressed in the form of PKM1 in normal cells and PKM2 in tumors. PKM2, a glycolytic pyruvate kinase isoenzyme, is expressed in many different cells, especially in tumor cells. Like HK2, PKM2 is an important rate limiting enzyme in glycolysis. PKM1 can form a stable tetramer with high enzyme activity, while PKM2 in tumor cells exists in the form of dimer with low enzyme activity, thus accumulating the upstream intermediate products of glycolysis into the anabolic pathway and promoting the growth and proliferation of tumor cells.

C-Myc is one of the core regulators of glycolysis [130]. In colorectal cancer, long non-coding RNA LINRIS blocks K139 ubiquitination of IGF2BP2, stabilizes the expression level of IGF2BP2 through autophagy lysosomal pathway, and promotes c-Myc mediated glycolysis through LINRIS-IGF2BP2-c-Myc axis, thereby promoting the progression of colorectal cancer [131]. In lung adenocarcinoma, the Wnt/β-catenin signaling can inhibit FTO expression, under the effect combined with YTHDF1, the increasing m6A level of c-Myc can promote tumor glycolysis and tumorigenesis [132].

In addition to these classic metabolic enzymes in glycolysis, m6A modification can also regulate some proteins related to glucose metabolism to participate in tumor progression. In GBM, as for temozolomide chemoresistance, JPX can promote the stability of PDK1 through m6A-dependent manner of FTO, thus promoting aerobic glycolysis and promote chemoresistance [133]. In cervical cancer and liver cancer, PDK4 is involved in glycolysis and ATP generation. YTHDF1/eEF-2 complex binding with IGF2BP3, can stabilize PDK4 through m6A manner, further promote glycolysis, and promote cancer progression [134]. C5aR1-positive neutrophils can promote glycolysis through up-regulating ENO1, which further promote breast cancer progression. In fact, C5aR1-positive neutrophils can secrete IL-1β and TNF-α to active ERK1/2 signaling, phosphorylate and stabilize WTAP, which can increase the m6A level of ENO1, and the up-regulated ENO1 can promote glycolysis, thus promoting tumor progression [135]. In renal cell carcinoma, the down-regulation of METTL14 can promote tumor cells metastasis. Due to METTL14 can negative regulate expression of BPTF through m6A manner, which activate enolase 2 and SRC proto-oncogene tyrosine kinase, thus can reprogram glycolytic to promote distal lung metastasis [136].

### The m6A and fatty acid metabolism

Lipid metabolism is a complex biochemical reaction in organism, including digestion, absorption, synthesis and decomposition of fat under different types of enzymes, which is essential for maintaining cell homeostasis. In cancer, because of the high demand for nutrients, tumor cells often regulate and utilize lipid metabolism to maintain their own proliferation, survival, invasion and metastasis. De novo synthesis of fatty acids is an important metabolic marker in cancer. Enhanced adipogenesis provides an important substance and energy source for tumor growth [137]. Fatty acid oxidation is an important source of cellular energy. Inhibition of fatty acid oxidation can inhibit the growth of tumor cells [138]. Up-regulation of FAO may maximize the production of ATP, reduce intracellular ROS and protect cancer cells from death [139]. Lipid transformation induces the activation key fatty acid synthesis enzymes in tumor cells, such as acetyl CoA carboxylase (ACC) and fatty acid synthase (FAS), and then promotes the proliferation and survival of cancer cells [140].

In hepatoma cells, FTO knockout significantly reduces the content of new adipogenic enzymes and intracellular lipids. This is because FTO knockout makes YTHDF2 play its recognition function, significantly increases the level of FASN m6A, decreases the stability and expression of FASN, and significantly decreases the protein levels of acetyl CoA carboxylase ACC and ATP citrate lyase, thus inhibiting the formation of new fat, leads to insufficient lipid accumulation and induces apoptosis [141].

Carnitine palmitoyltransferase 1B (CPT1B) is the rate limiting enzyme of fatty acid oxidation. High m6A methylation level in drug-resistant cells can trigger the splicing of ESRRG precurser, increases the expression of CPT1B and induces up-regulation of ERRγ in chemoresistant cells, which can promote fatty acid oxidation of tumor cells and enhance chemoresistance of tumor cells [142]. In esophageal cancer, m6A reader HNRNPA2B1 can up-regulate fatty acid metabolism-related genes ACLY and ACC1, and thus promote cellular lipid accumulation, which can further promote tumor progression, including proliferation, migration and invasion of cancer cells [143].

### The m6A and amino acid metabolism

Amino acids are produced by the hydrolysis of proteins. The metabolism of amino acids in the body includes two main aspects: one is the synthesis of proteins, peptides and other nitrogen-containing substances needed for their own synthesis; the other is the decomposition amino acids through deamination, transamination functions to produce α-ketoacid, CO2, etc. Among them, α-ketoacids can release energy through TCA oxidation [144]. Serine, glycine and other nonessential amino acids are closely related to the occurrence and development of cancer, so inhibiting the activity of these nonessential amino acids can be used as a potential means of cancer treatment. TCA is not only the final metabolic pathway of the three nutrients, but also the hub of carbohydrate, lipid and amino acid metabolism. In order to maintain a functional TCA cycle, cancer cells usually rely on the elevation of glutamatelyase. Therefore, glutamine decomposition is another important feature of tumor energy metabolism [145].

VHL protein is HIF family substrate recognition site, targeting HIF family can degrade ubiquitin mediated proteasome. In renal clear cell carcinoma, the lack of tumor suppressor gene VHL is a significant sign. VHL and FTO have the function of synthetic death. The inactivation of VHL leads to the structural activation of VEGF and PDGF, which can target downstream glutamine transporter SLC1A5, promotes the metabolic reprogramming of VHL deficient renal cancer cells, selectively reduces the growth and survival of VHL deficient renal cancer cells [146].

Long non-coding RNA Linc00266-1 can encode a peptide composed of 71 amino acids, which is called RNA binding regulatory peptide (RBRP). As long as the peptide mainly interacts with RNA binding proteins including IGF2BP1, Linc00266-1, as a subunit regulated by m6A reader, enhances the recognition of c-Myc m6A site by IGF2BP1, increases the mRNA stability and expression level of c-Myc and promotes the tumorigenesis [147].

Histone modifications have huge effects on gene expression. Huang et al. reported that histone H3 trimethylation at lysine 36 (H3K36me3) significantly enriched m6A modification, which can be recognized and bounded by METTL14. In the embryonic stem cells of mouse, the knockdown of METTL14 and depletion of H3K36me3 significantly reduces m6A abundance, and increases the stemness of cells [148]. ALKBH1 can also works as an m6A demethylation enzyme. In lung cancer, ALKBH1 is up-regulated, due to its function of demethylation the essential residues Y184, H231, D233, H287, R338 and R344, it can significantly promote tumor migration and invasion of cancer cells [149].

### The m6A and mitochondrial metabolism

As the energy factory of cells, mitochondria produce ATP to cells by burning glucose, lipids and amino acids, which can complete various life activities for cell functions. However, in recent years, studies have found that there are many correlations between mitochondrial metabolism and tumorigenesis. At the same time, because mitochondria are the metabolic center of cells, and tumor cells have the characteristics of abnormal metabolism, the development of compounds targeting mitochondria has become a new direction of anti-tumor research. Although the Warberg effect has been verified in all kinds of tumorigenesis, it has been found that oxidative phosphorylation and mitochondria dependent energy synthesis are the key processes to maintain the stemness of some tumor cells in recent years [150]. Many studies have found that the growth of tumor cells can be regulated through mitochondrial metabolism reprogram.

One carbon metabolism, including folate cycle, methionine cycle and sulfur transfer pathway, plays a key role in a series of metabolic processes required for tumor cell survival and growth. Methylenetetrahydrofolate dehydrogenase 2 (MTHFD2) is a mitochondrial enzyme involved in one carbon metabolism, which regulates HIF-2α transcriptomic mechanism thus influences the progression of RCC. Although MTHFD2 has not been identified as an m6A methylase by definition, MTHFD2 expression is significantly increased in renal cell carcinoma and is involved in regulation the overall level of m6A methylation, especially HIF-2α m6A methylation, promotes HIF-2α expression, thus promotes aerobic glycolysis of tumor cells and tumor progression. One carbon metabolism is associated with HIF-2α dependent metabolic reprogramming combined with the RNA methylation modification. MTHFD2 can regulate mRNA methylation and specifically increase the methylation level of METTL3 dependent HIF-2α. HIF-2α in turn can bind to the promoter region of MTHFD2 gene, and its overexpression increases the level of MTHFD2, thus forming a positive feed-forward loop to promote metabolic recombination and tumor progression [151].

Meclofenac is a non-steroidal anti-inflammatory drug, which is mainly used in the treatment of arthritis, analgesia and dysmenorrhea. However, recent studies have found that meclofenac can also be used as a highly selective FTO inhibitor to reduce ROS accumulation and apoptosis, and participates in the regulation of mitochondrial function [152]. FTO plays an anti-tumor role in renal clear cell carcinoma. Chronic mitochondrial dysfunction can lead to the loss phenotype of VHL (von Hippel Lindau), the most common mutated tumor suppressor gene in renal cell carcinoma. Accumulation of metabolites during mitochondrial dysfunction can inhibit the degradation of VHL dependent HIF-α, pseudo hypoxia state similar to the loss of VHL is formed. VHL deficient cells with FTO expression increase PGC-1α expression by reducing m6A methylation level, which can recovery mitochondrial activity, promote oxidative stress and ROS production, thus inhibiting tumor growth [153].

In breast cancer, tamoxifen-resistant cancer cells highly express adenylate kinase 4 (AK4) and METTL3. Inhibition of METTL3 in tamoxifen-resistant cancer cells can lead to the decrease of AK4, which further promotes the apoptosis of mitochondrial, thus attenuates resistance to tamoxifen [154].

## Clinical application of m6A methylation in cancer metabolism

At present, the related researches of m6A modification mainly focus on the basic research, but the related researches on its application in clinical transformation are very limited. Here, we summarized the basic experiments and signaling pathways related to anti-cancer therapy, especially in chemotherapy, radiotherapy and immunotherapy by targeting the m6A methylation in tumor. Just as Wang et al. predicted, the cancer therapies targeting m6A methylation might be a promising treatment options [30].

### Implication in chemotherapy

Chemotherapy is one of the most effective means to treat cancer. Together with surgery and radiotherapy, it is called the three major treatment means of cancer. The purpose of chemotherapy is to cure tumor, delay tumor metastasis, alleviate tumor symptoms and minimize toxic reactions.

Studies demonstrate that the m6A modification of genes show the relevance to chemotherapy in cancer. In pancreatic cancer, METTL3-depleted cancer cells show higher sensitivity to gemcitabine, 5-FU and cisplatin [39] (Table 3). This result shows that m6A methylation regulated cancer can play an important role in chemotherapy. In non-small cell lung cancer, in order to adapt hypoxia microenvironment, YTHDF1 is down-regulated, which further causes the resistance to cisplatin through regulating the translation of CDK2, CDK4 and cyclin D1 [155]. However, in colorectal cancer, YTHDF1 is significantly up-regulated, which reduces the sensitivity to cisplatin. Thus, YTHDF1 expression shows totally different expression models in distinct cancer. In fact, YTHDF1 can bind to the 3’-UTR of GLS1, and promote the protein synthesis of GLS1. Thus, YTHDF1 promotes cisplatin resistance through reprogramming GLS1-glutamine metabolism in colorectal cancer [156]. In leukemia, the resistance to tyrosine kinase inhibitor (TKI) is dependent on over-expression of FTO [157]. PARP inhibitor is an effectively targeting therapeutic drug in ovarian cancer, which mainly used to platinum sensitive recurrent ovarian cancer patients, thus the resistance to PARP inhibitor is a big challenge for ovarian cancer patients. The m6A methylation level can contribute to PPAR inhibitors resistance to the BRCA deficient cancer cells through up-regulating Wnt/β-catenin pathway via stabilizing FZD10. The increases of FZD10 m6A level, which mediated by down-regulation of FTO and ALKBH5, promotes PARP inhibitor resistance, which is due to the increase of homologous recombination activity [158]. In pancreatic ductal adenocarcinoma (PDAC), ALKBH5 works as a tumor suppressive gene, which shows more chemosensitizing to gemcitabine. In addition, The silencing of ALKBH5 significantly promotes proliferation, migration and metastasis of cancer cells [49].

**Table 3:** The relationship between m6A enzymes and cancer treatment

Classification	Cancer Type	M6A Related Enzymes	Biological Behavior Changes	References
Chemotherapy	Pancreatic cancer	METTL3	Decrease sensitivity to gemcitabine, 5-FU and cisplatin	[39]
	Non-small cell lung cancer	YTHDF1	Promote chemosensitivity to cisplatin	[155]
	Colorectal cancer	YTHDF1	Promote cisplatin resistance through reprogram GLS1-glutamine metabolism	[156]
	Leukemia	FTO	Promote resistance to tyrosine kinase inhibitor (TKI)	[157]
	Ovarian cancer	FTO; ALKBH5	Promote the sensitivity to PARP inhibitor resistance	[158]
	Pancreatic ductal adenocarcinoma	ALKBH5	Promote chemosensitize to gemcitabine	[49]
Immunotherapy	Colorectal cancer and melanoma	METTL3; METTL14; YTHDF2	Decrease the response of pMMR-MSI to PD-1 treatment	[159]
	Tumor	FTO	Resistance to toxic T cells	[161]
Radiotherapy	Pancreatic cancer	METTL3	Decrease the sensitivity to irradiation	[39]
	Lung adenocarcinoma	IGF2BP2/3	Decrease the harmful effects of radiation on lung adenocarcinoma	[106]
	Cervical squamous cell carcinoma	FTO	Enhance chemo-radiotherapy resistance	[162]
	GBMs	METTL3	Promote resistance of cancer cells to γ-irradiation	[163]

### Implication in immunotherapy

PD-1 is a star immunosuppressive molecule, which can down-regulate the response of the immune system, and regulates the immune system by inhibiting the activity of T cells, so as to promote immune tolerance. In tumors, PD-1 can help tumor cells escape from the immune system. At present, there are many monoclonal antibodies targeting PD-1 on the market and used in clinic. However, in some conditions, the response of tumor patients to anti-PD-1 therapy is limited, so it is necessary to combine with other targeted drugs. In colorectal cancer and melanoma, knockout of METTL3 and METTL14 inhibits m6A modification, stabilizes STAT1 and IRF1 mRNA expression, and promotes IFNγ-Stat1-Irf1 signaling transduction through the synergistic effects of YTHDF2, which increases the expression of CD8^+^ T cells, and increases the secretion of IFN-γ, CXCL9 and CXCL10, enhances the response of pMMR-MSI-L to PD-1 treatment [159].

In human acute myeloid leukemia (AML), FTO promotes the occurrence of leukemia. FB23 and FB23-2, as the inhibitors of FTO, can directly bind with FTO to selectively inhibit their expression enzyme activity, and then inhibiting the proliferation of cells and inducing their differentiation or apoptosis. In xenotransplantation mice, FB23-2 also increases the abundance of m6A and inhibits the progression of tumor [160]. In addition, Su et al. reported two other small molecular inhibitors targeting FTO, CS1 and CS2, which also play an anti-tumor role. They found that FTO can also regulate the immune microenvironment of tumor by regulating m6A methylation, inhibiting the expression of FTO through CS1 or CS2, and inhibiting the expression of immune checkpoint genes, such as leukocyte immunoglobulin like receptor B4 (LILRB4), which makes tumor cells more sensitive to toxic T cells, and significantly reduced the self-renewal of cancer cells by reprogramming immune response [161].

### Implication in radiotherapy

Radiotherapy plays an increasingly prominent role in tumor treatment and has become one of the main means of treating malignant tumors. As we all known, the curative effect of radiotherapy depends on its radiosensitivity. Recently, studies show that m6A modification participates in the regulation of radiosensitivity.

In pancreatic cancer, METTL3-depleted cancer cells showed higher sensitivity to the irradiation [39]. In lung adenocarcinoma, radiation causes the reduction expression of miR-29b-3p, which targets VANGL1, thus increasing the expression of VANGL1 in cancer. The deletion of IGF2BP2/3 in m6A reader destroys the stability and expression of VANGL1 mRNA after radiation. Knockout VANGL1 can harm DNA from damage by activating BRAF-TP53BP1-Rad51 cascade, enhances the harmful effects of radiation on lung adenocarcinoma [106]. In cervical squamous cell carcinoma, FTO reduces β-catenin transcript, and promotes ERCC1 activity, thus enhancing chemo-radiotherapy resistance of cancer cells [162]. In GBMs, METTL3 participates in the GSC maintenance and dedifferentiation by targeting SOX2, which enhances the stability of SOX2. Therefore, silence METTL3 can reduce the expression of SOX2, which further enhance the sensitivity of cancer cells to γ-irradiation [163].

## Conclusion

Over the years, researchers have been working on whether tumor is a genetic disease or a metabolic disease. However, the molecular mechanism of tumor and metabolic reprogramming is very complex. In this review, we comprehensively analyze the important role of m6A methylation as a regulator of tumor metabolism. At present, the interaction between m6A methylation and tumor metabolism is very complex. On one hand, tumor metabolic stress can regulate the abnormal m6A methylation. On the other hand, the maladjustment of m6A methylation can regulate the signaling pathway, transcription factors and metabolic enzymes related to metabolic recombination. Currently, the researches on m6A methylation mainly focus on the regulation of related signaling pathways and transcription factors. However, the researches on metabolic enzymes, including glucose, lipid acid and amino acid metabolic enzymes, still need further investigate. Take Warburg effect, one of the most important characters in cancer as an example. As shown in the previous studies, Warburg effect can be regulated by m6A methylase (such as METTL3), m6A demethylase (such as ALKBH5) and m6A readers (such as IGF2BP2) through regulating the expression of various signaling pathways and molecules. The interaction between m6A methylation and Warburg effects is mutual, and the complex mechanisms need to be further explored and clarified. In fact, there is still a long way to go in the study of tumor metabolism. In conclusion, in the process of tumor cell metabolism reprogramming, m6A methylases and its processes are important regulatory factors. It is important that specific drugs and small molecular inhibitors targeting m6A methylase and signaling pathways have been carried out in vitro and in animal experiments. Further researches and clinical experiments of m6A methylases-related drugs are the new directions of tumor treatment in the future.

## Supplementary Information

**Additional file 1: Table 1.** The metabolism-related pathways in cancer with m6A enzymes. **Table 2.** Metabolism-related transcription factors with m6A enzymes. **Table 3. **The metabolism-related non-coding RNA in cancer with m6A enzymes.

## Data Availability

All the data obtained and/or analyzed during the current study were available from the corresponding authors on reasonable request.

## Abbreviations

18F-FDG: 18F-fluorodeoxyglucose
ACC: Acetyl CoA carboxylase
ALKBH5: AlkB homolog 5
AML: Acute myeloid leukemia
AMPK: 5’-AMP-activated protein kinase
ATG: Autophagy related genes
ATP: Adenosine triphosphate
CDS: Sequence coding for aminoacids in protein
CK2: Casein kinase II
CPT1B: Carnitine O-palmitoyltransferase 1B
CTNNB1: Catenin beta 1
CXCL: C-X-C motif ligand
CXCR4: C-X-C motif receptor 4
DDX3: DEAD box helicase 3 X-linked
DGCR8: DGCR8 microprocessor complex subunit
ECAR: Extracellular acidification rate
EGFR: Epidermal growth factor receptor
EMT: Epithelial-mesenchymal transition
ERK: Extracellular regulated protein kinases
ESRRG: Estrogen-related receptor gamma
FAO: Fatty acid oxidation
FASN: Fatty acid synthase
FOXA2: Forkhead box A2
FOXM1: Forkhead box M1
FTO: Fat mass and obesity associated
G6PD: Glucose-6-phosphate dehydrogenase
GAS5: Growth arrest-specific 5
GLS2: Glutaminase 2
GLUT: Glucose transporter
GPER: G protein coupled estrogen receptor
HBXIP: Hepatitis B virus X-interacting protein
HCC: Hepatocellular carcinoma
HDAC3: Histone deacetylase 3
HIF: Hypoxia-inducible factor
HK2: Hexokinase 2
hnRNPA2B1: Heterogeneous nuclear ribonucleoprotein A2/B1
HOXB13: Homeobox B13
HSP: Heat shock protein
IFN: Interferon
IGF2BP: Insulin-like growth factor 2 mRNA-binding protein
IRF1: Interferon regulator factor 1
JNK: C-jun N-terminal kinase
MALAT1: Metastasis associated lung adenocarcinoma transcript 1
MAPK: Mitogen-activated protein kinases
MCM5: Minichromosome maintenance complex component 5
METTL14: Methyltransferase Like 14
METTL16: Methyltransferase Like 16
METTL3: Methyltransferase Like 3
MMP: Matrix metallopeptidase
MTHFD2: Methylenetetrahydrofolate dehydrogenase 2
mTOR: Mechanistic target of rapamycin
NADPH: 2,4-Dienoyl CoA reductase 1
NF-κB: Nuclear factor-κB
NANOG: Nanog homeobox
OCT4: POU class 5 homeobox 1
OXPHOS: Oxidative phosphorylation
P2RX6: Purinergic receptor P2X ligand gated ion channel 6
PADI2: Peptide arginine deaminase II
PD-1: Programmed cell death protein 1
PDGF: Platelet derived growth factor
PDK2: Pyruvate dehydrogenase kinase 2
PHLPP2: PH domain and leucine rich repeat protein phosphatase 2
PI3K: Phosphatidylinositide 3-kinases
PK: Pyruvate kinase
PPAR: Peroxisome proliferator activated receptor
PRR5: Proline rich 5
PTEN: Phosphatase and tensin homolog
RBM15: RNA binding motif protein 15
RBRP: RNA binding regulatory peptide
ROS: Reactive oxygen species
SLC1A5: Solute carrier family 1 member 5
SOX2: Sex determining region Y-box 2
SPRED2: Sprouty-related EVH1 domain containing 2
SRF: Serum response factor
STAT: Signal transducer and activator of transcription
SUMO: Small ubiquitin-like modifier
TAZ: Tafazzin
TCA: Tricarboxylic acid
TLR4: Toll-like receptor 4
TMZ: Temozolomide
TNF: Tumor necrosis factor
TRIM25: Tripartite motif containing 25
UBC9: Ubiquitin-conjugating enzyme E2I
UTR: Untranslated region
VEGF: Vascular endothelial growth factor
VHL: Von Hippel-Lindau tumor suppressor
VIRMA: Vir like m6A methyltransferase associated
WIF-1: Wnt inhibitor factor-1
WTAP: Wilms tumor 1 associated protein
XIST: X-inactive specific transcript
YAP: Yes-associated protein
YTHDC: YTH domain-containing
YTHDF: YTH domain-containing family protein
