# Supplementary material for: The role of m6A RNA methylation in cancer metabolism
Source: Mol Cancer. 2022 Jan 12;21:14. doi: 10.1186/s12943-022-01500-4 (PMC8753874; doi:10.1186/s12943-022-01500-4)
Supplement: Supplementary file 1 — Additional file 1: Table 1. The metabolism-related pathways in cancer with m6A enzymes. Table 2. Metabolism-related transcription factors with m6A enzymes. Table 3. The metabolism-related non-coding RNA in cancer with m6A enzymes. [file 12943_2022_1500_MOESM1_ESM.docx]

***Supplementary Table 1 The metabolism-related pathways in cancer with m6A enzymes***

| ***Classification*** | ***Cancer Type*** | ***M6A Enzymes*** | ***Biological Behavior Changes of Cancer Cells*** | ***Related Pathways*** | ***References*** |
| --- | --- | --- | --- | --- | --- |
| *Pathways* | *Retinoblastoma* | *METTL3* | *Promote the proliferation, migration and invasion of cancer cells* | *mTOR* | *[33]* |
|  | *Endometrial cancer* | *METTL3; METTL14; YTHDF1; YTHDF2* | *Inhibit the proliferation and tumorigenicity of cancer cells* | *mTORC2* | *[34]* |
|  | *Gastric cancer* | *FTO* | *Inhibit the anti-tumor effects of drugs like 5-FU, DDP and TAX* | *mTORC1* | *[36]* |
|  | *Pancreatic cancer* | *METTL3* | *Resistance to chemotherapy and radiotherapy* | *MAPK* | *[39]* |
|  | *Renal cell carcinoma* | *METTL14* | *Decrease the invasion and migration ability of cancer cells through increasing P2X6 mRNA splicing* | *ERK1/2* | *[40]* |
|  | *Colorectal cancer* | *METTL3* | *Promote the invasion and metastasis of tumor cells* | *MAPK* | *[41]* |
|  | *Bladder cancer* | *METTL3* | *Promote the proliferation of cancer cells* | *PTEN* | *[42]* |
|  | *Hepatoblastoma* | *METTL3* | *Activate Wnt-β-Catenin signaling pathway and promote tumor progression* | *Wnt-β-Catenin* | *[47]* |
|  | *Metastatic endometrial carcinoma* | *FTO; YTHDF2* | *Activate Wnt signaling pathway and promote tumor invasion and metastasis* | *HOXB13* | *[48]* |
|  | *Pancreatic ductal adenocarcinoma* | *ALKBH5* | *Regulate Wnt-β-Catenin signaling and inhibit drug resistance of cancer cells* | *WIF1* | *[49]* |
|  | *Prostate cancer* | *METTL3* | *Regulate Hedgehog pathway and inhibit tumor cells apoptosis* | *Gli1* | *[52]* |
|  | *Ovarian cancer* | *ALKBH5* | *The activated NF-κB up-regulate ALKBH5 and promote the occurrence of ovarian cancer* | *NANOG* | *[55]* |
|  | *Bladder cancer* | *METTL3* | *Promote the malignant biological behaviors of cancer cells* | *NF-κB-MYC* | *[57]* |
|  | *Endometrial carcinoma* | *WTAP* | *Activate NK-κB signaling and promote the proliferation, migration and invasion of cancer cells* | *CAV-1* | *[58]* |

***Supplementary Table 2 Metabolism-related transcription factors with m6A enzymes***

| ***Classification*** | ***Cancer Type*** | ***M6A Enzymes*** | ***Biological Behavior Changes of Cancer Cells*** | ***Related Transcription factors*** | ***References*** |
| --- | --- | --- | --- | --- | --- |
| *Transcription factors* | *Breast cancer* | *ALKBH5* | *HIF-1α and HIF-2α activate ALKBH5 and promote the phenotype of breast cancer stem cells* | *NANOG* | *[63]* |
|  | *Hepatocellular carcinoma* | *YTHDF1* | *Promote autophagy and malignant progression of tumor cells* | *ATG2A; ATG14* | *[64]* |
|  | *Hepatocellular carcinoma* | *METTL3* | *Promote metabolic reprogramming and malignant biological behaviors* | *HIF-1α* | *[65]* |
|  | *Glioblasoma carcinoma* | *ALKBH5* | *Promote the stemness and proliferation of cancer cells* | *FOXM1* | *[69]* |
|  | *Oral squamous cell carcinoma* | *ALKBH5* | *Promote the chemotherapy resistance of cancer cells* | *FOXM1* | *[70]* |
|  | *Colon cancer* | *METTL3* | *Promote drug resistance of cancer cells* | *P53* | *[76]* |
|  | *Non-small cell lung cancer* | *METTL3; YTHDF1/3; eIF3b* | *Promote the invasion, metastasis and drug resistance of cancer cells* | *YAP* | *[80]* |
|  | *Colorectal cancer* | *YTHDF3* | *Inhibit the progression* | *YAP* | *[81]* |
|  | *Non-small cell lung cancer* | *ALKBH5; YTHDF1-3* | *Inhibit the growth and metastasis of tumor cells* | *YAP* | *[82]* |
|  | *Lung cancer* | *METTL3* | *Promote the proliferation, invasion and migration of cancer cells* | *c-Myc* | *[85]* |
|  | *Oral squamous cell carcinoma* | *METTL3; YTHDF1* | *Inhibit the malignant progression of cancer cells* | *c-Myc* | *[86]* |
|  | *Gastric cancer* | *FTO* | *Inhibit the invasion and migration of cancer cells* | *c-Myc* | *[87]* |
|  | *Gastric cancer* | *METTL3* | *Target MCM5 and MCM6 thus promoting tumor progression* | *c-Myc* | *[48]* |
|  | *Acute myeloid leukemia* | *METTL14* | *Inhibit the terminal differentiation and promote the proliferation of AML cells* | *MYB-MYC* | *[88]* |
|  | *Hepatoma cells* | *YTHDF2* | *Regulate the stemness of cancer cells* | *OCT4* | *[96]* |
|  | *Endometrial cancer* | *IGF2BP1* | *Promote the progression of cancer* | *SOX2* | *[98]* |
|  | *Colorectal cancer* | *METTL3; IGF2BP2* | *Promote the progression of cancer* | *SOX2* | *[99]* |
|  | *Bladder cancer* | *METTL3* | *Promote the self-renewal of cancer stem cells* | *SOX2* | *[100]* |
|  | *Hepatocellular carcinoma* | *WTAP* | *Regulate p21-p27 axis to regulate the progression of cancer cells* | *ETS1* | *[103]* |
|  | *Hepatocellular carcinoma* | *METTL3* | *Promote the progression of cancer* | *Snail* | *[105]* |

***Supplementary Table 3 The metabolism non-coding RNA in cancer with m6A enzymes***

| ***Classification*** | ***Cancer Type*** | ***M6A Enzymes*** | ***Biological Behavior Changes of Cancer Cells*** | ***Related RNAs*** | ***References*** |
| --- | --- | --- | --- | --- | --- |
| *Non-coding RNAs* | *Bladder cancer* | *METTL3* | *Promote the progression and shows poor prognosis* | *miR-221/222* | *[42]* |
|  | *Colorectal cancer* | *METTL14* | *Inhibit the invasion and migration of cancer cells* | *miR-375* | *[110]* |
|  | *Metastatic hepatocellular carcinoma* | *METTL14* | *Enhancement of invasion ability of tumor cells* | *miR-126* | *[112]* |
|  | *Non-small cell lung cancer* | *METTL3; YTHDF1/3* | *Promote the invasion and metastasis of cancer cells* | *MALAT1* | *[80]* |
|  | *Thyroid cancer* | *IGF2BP2* | *Promote the proliferation and invasion of cancer cells* | *MALAT1* | *[114]* |
|  | *Colorectal cancer* | *METTL14; YTHDF2* | *Inhibit the proliferation and invasion of tumor cells* | *XIST* | *[116]* |
|  | *Glioma* | *ALKBH5* | *Promote TMZ resistance in cancer cells* | *Circ-0072083* | *[117]* |
|  | *Non-small cell lung cancer* | *IGF2BPs* | *Inhibit the proliferation and metastasis of cancer cells* | *Circ-NDUFB2* | *[118]* |
|  | *Hepatocellular carcinoma* | *METTL3; METTL14* | *Activate Wnt/β-catenin pathway and induce sorafenib resistance* | *Circ-SORE* | *[119]* |
